# Cannabis Use and Parenting Practices among Young People: The Impact of Parenting Styles, Parental Cannabis-Specific Rules, and Parental Cannabis Use

**DOI:** 10.3390/ijerph19138080

**Published:** 2022-07-01

**Authors:** Karmen Osterc Kokotovič, Mateja Pšunder, Andrej Kirbiš

**Affiliations:** 1 National Institute of Public Health, 2000 Maribor, Slovenia; 2 Department of Pedagogy, Faculty of Arts, University of Maribor, 2000 Maribor, Slovenia; mateja.psunder@um.si; 3 Department of Sociology, Faculty of Arts, University of Maribor, 2000 Maribor, Slovenia; andrej.kirbis@um.si

**Keywords:** parenting styles, parental cannabis-specific rules, parental cannabis use, adolescents, substance use

## Abstract

Cannabis is the most commonly used illicit drug. Its use typically starts in adolescence, and parents play a key role in young people’s cannabis use. Our study aimed to examine and compare the effects of parenting styles, parental cannabis-specific rules, and parental cannabis use on young people’s cannabis use. The research sample consisted of 839 students from various secondary education programs in Slovenia, aged 14 to 21. Associations between the young people’s lifetime cannabis use and their experience of parenting practices were assessed using logistic regression, with demographic, socioeconomic, educational, health, and risk behaviors controlled in a multivariate model. Maternal authoritative parenting (in comparison with permissive parenting), strict maternal, cannabis-specific rules, and parental cannabis non-use statistically significantly reduced the likelihood of young people’s cannabis use. Its strongest predictor was parental cannabis use, followed by the mother’s specific cannabis-use rules and maternal parenting style. The findings of our study can contribute to the development of public health policies to more effectively prevent cannabis use among adolescents and emerging adults, including by designing prevention programs aimed at strengthening parents’ general and cannabis-specific practices and competences.

## 1. Introduction

Cannabis is the most commonly used illicit drug and is about five times more prevalent than other illicit drugs [1]. Cannabis use typically starts in early adolescence [2], and as a result, young people’s use of cannabis is a major public health concern [1]. Furthermore, the data on cannabis use in the general population (15–64 years) show that cannabis use is most prevalent among young people aged 15–24 years [1]. Cannabis is the most commonly used illicit drug among the secondary school population in Slovenia [3,4]; the data from the European School Survey Project on Alcohol and Other Drugs (ESPAD) in Slovenia show that 23.2% of adolescents aged 15 and 16 years have tried cannabis at least once [3]. These data put Slovenia above the average among the European countries participating in the ESPAD survey, with the European prevalence being 16% in 2019. The data from the 2018 Health Behavior in School-aged Children survey (HBSC) for Slovenia, which included 11-, 13-, 15- and 17-year-olds, show that cannabis was the most commonly used illicit drug among 17-year-olds (42.5% had used it at least once in their lifetime) [4]. A special HBSC COVID-19 survey in 2020 similarly showed that 45.2% of Slovenian 18-year-olds reported having used cannabis at least once in their lifetime, 38.3% in the last 12 months, and 21.2% in the last 30 days. Daily cannabis use was reported by 3.7% of 18-year-olds [5].

Cannabis use, especially among adolescents, has serious adverse effects [6]. Adolescents are much more likely to engage in delinquent behavior and criminal activity as a result of its use [6,7], thus risking developmental problems [8,9,10]. Adolescents who start using cannabis before the age of sixteen have more cognitive problems than those who start using cannabis after the age of sixteen [9]. Similarly, researchers have found cognitive deficits in young adults who were regular cannabis users in their youth [11]. Adolescence is a period of neuromaturation, and there is increasing evidence that the adolescent brain is more sensitive to the effects of drugs than the adult brain [12]; thus, the adverse consequences of drug use in young people are greater than among adults [13].

Various factors have been found to influence adolescents’ drug use, including individual risk and deviant behaviors (e.g., aggressive behavior), poor mental health and emotional distress, inadequate coping strategies, poor school outcomes (e.g., low academic performance and school absenteeism), and problematic peer group characteristics (e.g., peer substance use) [14,15,16,17]. In addition, the adolescent’s family is also found to be vital in reducing adolescent substance use, including cannabis use [2]. Several studies confirm that parents have an important influence on young people’s cannabis use through their parenting styles [18,19,20,21,22,23,24,25], their attitudes towards and rules about drug use [26,27,28], and their own behavior, i.e., whether parents use drugs [26,29,30,31,32,33,34]. For effective intervention and prevention of young people’s drug use, it is critical to understand the role played by these parental factors.

### 1.1. Parenting Styles and Drug Use

**Figure 1:**
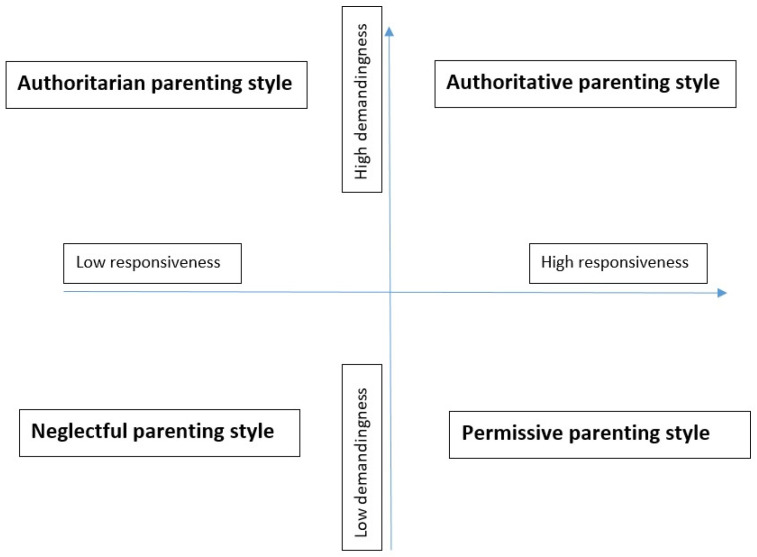
The Cartesian plane depicting parenting styles.

The family’s role in preventing drug use is manifested, among other things, through parenting styles, which may increase or decrease the risk of drug use [35]. Young people’s perceptions of their parents’ parenting styles are formed relatively early in life and remain stable over time [21]. Research examining the relationship between parenting styles and youth outcomes has traditionally followed the typology of four parenting styles [23], which is based on a combination of two dimensions of parenting: parental responsiveness, which refers to parental support, care, love, encouragement, and understanding of the child, and parental demandingness, which encompasses those parenting practices that direct, monitor, and control the child’s behavior. The combination of the two dimensions forms four parenting styles: authoritative (high responsiveness and high demandingness), authoritarian (low responsiveness and high demandingness), permissive (high responsiveness and low demandingness), and neglectful (low responsiveness and low demandingness) [18,36]. Parenting styles and two parenting dimensions may be depicted diagrammatically using a Cartesian plane (Figure 1). Studies show that parenting styles predict children’s well-being in the areas of social competence [37,38], academic achievement [39,40,41], psychosocial development [42], and problem behavior [40,43], including drug use [23,25,44].

The authoritative parenting style (high responsiveness and high demandingness) has been shown to be the most effective parenting style when it comes to protecting adolescents from drug use [6,21,25,44,45,46]. Adolescents with authoritative parents tend to make fewer attempts to use cannabis than adolescents with permissive, authoritarian, and neglectful parents. The highest number of cannabis use attempts is among adolescents with neglectful parents [21].

Research on authoritarian and permissive parenting styles, however, has yielded inconsistent results [6,44,45]. The data from the 2013 Slovenian Youth Survey, which covered young people aged 16–27, showed that the permissive and authoritarian parenting styles were associated with more frequent use of drugs, including cannabis [25]. On the other hand, some studies [20,24,43] have found no statistically significant differences between authoritative and authoritarian parenting styles regarding drug use. It is possible that both reduce cannabis use to a similar extent because of the dimension of demandingness present in both parenting styles, which reflects parental control [22,45]. Results of studies on the permissive parenting style are also mixed; some studies have shown that the permissive parenting style is a risk factor for adolescent drug use [21,24,25,27], while research in the European context [23] has shown that the permissive parenting style, similarly to authoritative parenting style, is a protective factor against drug use.

It is possible that both authoritative and permissive parenting reduce the risk of drug use because both have a high responsiveness dimension. Parental responsiveness, manifested by an accepting and supportive attitude towards the child, has been associated with lower cannabis use in adolescents [47,48,49,50]. However, the dimension of demandingness, reflected in parental supervision, monitoring, and direction of the child, present to the largest extent among authoritative and authoritarian parenting, is also associated with lower adolescent cannabis use [48,51,52,53,54,55,56]. Parental monitoring, and supervision, knowing where the adolescent is, what the adolescent is doing, and whom the adolescent is associating with are the most effective ways to prevent adolescent cannabis use [6].

### 1.2. Specific Parenting Practices Regarding Young People’s Drug Use

Social development theory suggests that adolescents learn behavioral patterns such as drug use [34] through models in the context of primary socialization [57]. Parents have an essential influence on drug use through their attitudes towards drug use [27], parenting practices such as monitoring their offspring’s activities [48,58,59], and their own drug use [30,31,32,33,60]. Children of parents who use cannabis are more likely to use cannabis themselves [60,61,62]. Young people’s drug use is also influenced by their perception of how parents would react if they found out about their substance use. The greater the child’s belief that they would be punished by parents for using cannabis or other substances, or that parents implement a “no tolerance” rule for adolescent drug use, the lower their use of cannabis [33,60,63]. On the other hand, adolescents who have already tried tobacco, alcohol, and cannabis perceive greater tolerance of drug use in their parents [27,64].

### 1.3. The Present Study

Our study aims to examine and compare the effects of parenting styles, parental cannabis-specific rules for their children’s cannabis use, and parental cannabis use on adolescent cannabis use. The present study extends the existing research in several ways: To our knowledge, our study is the first to compare the role of general parenting styles, parental cannabis-specific rules, and parental cannabis use separately for mothers and fathers. Second, we built on a similar study of adolescents in the Netherlands [33], where multivariate models indicated that lifetime cannabis use was linked to parental support, demandingness, and parental cannabis use, but not to parental cannabis-specific rules. However, our study examines a combination of parenting dimensions in the form of the standard typology of parenting styles. Third, our study is the first to compare the impact of three parenting practices on young people’s cannabis use in a non-Western European country. Although the authoritative parenting style has typically been recognized as the most beneficial for reducing substance use [20,21], evidence on the impact of parenting styles on youth outcomes in different countries is needed, since an increasing number of studies indicate that optimal parenting styles may vary across cultures [65,66,67]. In addition, recent evidence from Europe suggests that compared with authoritative parenting, permissive parenting has become as beneficial or more beneficial for youth outcomes, including being protective against youth substance use [23,68,69]. Adding to the literature base to include evidence from a post-socialist country serves to extend and deepen knowledge of parent-youth relationship processes and cannabis use protective factors. Finally, we controlled for several confounding variables in the domain of demographic, socioeconomic, family, and health factors, and the use of another substance (alcohol), in order to tease out the effect of three parenting practices on the cannabis use of adolescents (we note that the term “adolescent” typically refers to young people between ages 10 and 18 [70] or 19 [71]. Although our sample included 14–21-year-olds, we use the term “adolescent” for the sake of brevity in the rest of the paper, since 98% of surveyed secondary school students in our sample were 19 or younger.).

In summary, the present study addressed the following research questions:

(1) Which maternal and paternal parenting style is the most protective against adolescent cannabis use, and which increases it?
(2) Which maternal and paternal parenting practices show the strongest associations with cannabis use: parenting styles, parental cannabis-specific rules, or parental cannabis use?
(3) Do maternal or paternal parenting practices play a greater role in adolescent cannabis use?

## 2. Materials and Methods

### 2.1. Sampling Strategy and Survey Procedure

The study followed the methodology of two international surveys (ESPAD and HBSC Behavior). The official national secondary school enrollment data for the academic year 2019/2020 represented the sample framework of the survey and the basis for the formation of a representative sample, using two-stage stratified sampling. In the first stage, we first sampled secondary schools, and in the second stage, secondary schools according to the program they implement (vocational education; technical education; and general education (gymnasium) [4]. The sampling procedure resulted in thirteen randomly selected secondary schools and 59 classes in total, in accordance with official school enrolment data. Students were asked to respond to an online survey. One thousand one hundred and thirty-six students aged 14 to 21 years opened the survey from September to November 2021, and 900 students (79.23%) completed the survey. Of all young people who completed the survey, 839 were included in the analyses (those living with both parents/caregivers and with no missing responses for other independent and confounding variables, and the outcome variable). Sample characteristics and predictor variables are described in Table 1.

The survey was conducted using the 1KA online survey tool. Secondary school counsellors were provided with a link to access the survey. The survey was conducted during class, under the supervision of the teacher to whom the survey was entrusted. All students were sent a link to their email address by the school so they could complete the survey on a computer or mobile device. The study followed the basic principles of the code of ethics in public health and social science research. All participants were informed about the content, aim, and procedure of the study. Their anonymity and personal data protection were guaranteed, and no personal identification data were collected. The schools and parents were informed that the data collected in the survey would be used for research purposes only. Students who did not wish to participate could refuse to.

### 2.2. Measures

For the purposes of the study, we analyzed data on the adolescents’ cannabis use, parental cannabis use, adolescents’ perception of the parents’ parenting style, cannabis-specific rules for the adolescents’ cannabis use, and several confounding variables.

The dependent variable, i.e., cannabis use at any time in life, was measured by asking adolescents whether they had ever used cannabis in their life based on the seven responses offered (1 = never, 2 = 1–2 times, 3 = 3–5 times, 4 = 6–9 times, 5 = 10–19 times, 6 = 20–39 times, 7 = 40 times or more). Since we were interested in lifetime cannabis use prevalence, the variable was dichotomized (1 = not used; 2 = used) [33].

Parenting styles were measured using the dimensions of parental responsiveness and parental demandingness. For the dimension of parental responsiveness, we used an abridged version of the Warmth/Affection Scale (WAS) [72], which contains eight items. The WAS is proven to be a reliable instrument [23] and tells us to what extent adolescents perceive their parents as loving, responsive, and involved. The adolescents completed two versions of the WAS, one for the perception of their mother’s responsiveness (Cronbach’s alpha = 0.89), and the other for the perception of their father’s responsiveness (Cronbach’s alpha = 0.92). The dimension of parental demandingness was measured using the Parental Control Scale (PCS) [73,74]. The PCS assesses how the adolescent perceives parental control over his or her behavior. The adolescents rated their perceived control separately for their father (Cronbach’s alpha = 0.81) and their mother (Cronbach’s alpha = 0.79), using the 13-item PCS scale. Adolescents rated individual items from both scales using a four-point scale (1 = almost never true, 4 = almost always true). Both parenting indexes (responsiveness and demandingness) measure parenting behavior (see [19,40]), so that higher scores on the scale indicate greater parental responsiveness and higher parental demandingness [74]. Following the models of S. Lamborn et al. [19] and Steinberg [75], we determined four parenting styles: authoritative, authoritarian, permissive, and neglectful, defined by the median (50th percentile) for each dimension. Authoritative mothers and authoritative fathers scored above the 50th percentile on both dimensions. Authoritarian mothers and authoritarian fathers scored above the 50th percentile on the demandingness dimension and below the 50th percentile on the responsiveness dimension. Permissive mothers and permissive fathers scored above the 50th percentile on the responsiveness scale and below the 50th percentile on the demandingness scale. Mothers and fathers scoring below the 50th percentile on both dimensions were categorized as neglectful.

Cannabis-specific rules concerning their adolescent’s cannabis use were tapped by asking the adolescents what they thought their mother’s or father’s reaction would be if they used cannabis. The adolescents chose one of five answers, separately for their father and their mother (1 = he/she would not allow (he/she does not allow), 2 = he/she would try to talk me out of it (he/she is trying to talk me out of it), 3 = he/she would not care (he/she does not care), 4 = he/she would approve (he/she approves), and 5 = I don’t know). The answers were dichotomized (1 = would not allow, 2 = other), since earlier research suggests that even a hint of parental tolerance of their children’s cannabis use increases the odds of young people’s cannabis use [33], and that a “no tolerance” rule is the most effective strategy to reduce tobacco, alcohol, and cannabis use [63].

Information on parental cannabis use was obtained by asking, “How often (if at all) do you think your mother or father uses cannabis?” The adolescents’ answers (never, a few times a year, once a month, 2–3 times a month, 1–2 times a week, 3–6 times a week, and every day) were used to identify the use of cannabis separately for the father and the mother. To examine the role of parental lifetime cannabis use prevalence, the variable was dichotomized (1 = does not use, 2 = uses).

Finally, we controlled for several confounding variables in multivariate models: sex (1 = male; 2 = female), age (in years), size of residential settlement (1 = rural; 2 = 1000–50,000 inhabitants; 3 = more than 50,000 inhabitants), educational track (1 = vocational secondary or lower, 2 = technical secondary or grammar school, 3 = college or higher), father’s and mother’s education (1 = vocational secondary or lower, 2 = technical secondary or grammar school, 3 = college or higher), self-assessed family material status (1 = below average; 2 = average; 3 = above average), self-rated health (1 = very poor or poor; 2 = fair, good or very good), academic achievement (1 = fail or satisfactory; (very) good or excellent), and alcohol use (1 = does not use, 2 = uses).

### 2.3. Statistical Analysis

The data collected through the online survey were processed using the IBM SPSS 28 statistical software. Descriptive statistics of frequencies (n) and percentages (%) were used. Lifetime prevalence of cannabis use was estimated using the percentage (%) and its corresponding 95% confidence interval (CI) (Table 1). Chi-square analysis was performed to determine the association between lifetime prevalence of cannabis use with all independent and confounding variables. Next, the associations between cannabis use prevalence, and the independent and confounding variables were tested with simple logistic regression to obtain the crude odds ratio (OR). Variables with *p* < 0.05 were included in multiple logistic regression analysis to obtain the final model and the adjusted OR (aOR) after controlling for potential confounders (Table 2) (The only examined potential confounder not significantly linked to cannabis use in the simple logistic regression model was self-rated health and was thus not included in the multivariate logistic model).

## 3. Results

The survey results are based on data provided by the adolescents who completed the survey on both parents. There were 839 of them in the sample (45.9% males and 54.1% females), of whom 25.7% reported having used cannabis in their lifetime. Table 1 shows the description of the sample according to the parenting variables studied and confounders. The results indicate that among adolescents with authoritative mothers, 13.6% have previously used cannabis in their life, while 26.9% of adolescents with authoritarian mothers, 31.0% of adolescents with permissive mothers, and 32.5% of adolescents with neglectful mothers have used cannabis in their life. Among adolescents with authoritative fathers, 16.1% have previously used cannabis. In comparison, 32.2% of adolescents with authoritarian fathers, 28.4% of adolescents with permissive fathers, and 27.1% of adolescents with neglectful fathers have used cannabis in their lifetime. Chi-square analyses indicated significant differences between maternal and paternal parenting styles and adolescent cannabis use (*p* < 0.001).

Among adolescents whose mothers would or do not allow them to use cannabis, 21.4% had used cannabis, compared with 42.5% of adolescents with more tolerant maternal cannabis-specific rules. Moreover, 22.2% of adolescents used cannabis among those whose fathers have strict cannabis-specific rules, compared with 39.8% of adolescents with more tolerant fathers. In addition, maternal cannabis use was linked to a higher proportion of adolescent users (81.0%), compared with adolescents whose mothers had not used cannabis (24.3% of adolescent users). Similarly, 78.4% of adolescents were cannabis users in a group whose fathers used cannabis, compared with 23.3% of adolescents whose fathers had not used cannabis.

Table 2 shows the results of the associations between the dependent variable (adolescents’ lifetime cannabis use) and all the predictor variables included in the multiple logistic regression model.

Based on the results of simple logistic analyses (not shown), self-rated health was not included in the multiple logistic regression due to non-significance (*p* > 0.05), while other confounders were included in the multivariate model. The results in Table 2 show that parenting styles were largely not a statistically significant predictor of adolescent cannabis use when controlling for other parenting practices and confounders. Compared with authoritative parenting, the mother’s permissive parenting more than doubled the odds of adolescent cannabis use (aOR = 2.00, 95% CI 1.10–3.65). The remaining two maternal parenting styles also increased the odds of adolescent cannabis use, but the impact did not reach statistical significance in the adjusted model (*p* > 0.05). As for paternal parenting, all three parenting styles increased the likelihood of adolescent cannabis use compared with the authoritative parenting style, but none reached statistical significance (*p* > 0.05).

Parental cannabis-specific rules concerning adolescent cannabis use were also a statistically significant predictor, but only those of the mother, not the father. If the mother does not have a strongly negative attitude towards her child’s cannabis use, the odds of the adolescent using cannabis in their lifetime almost tripple (aOR = 2.81, 95% CI 1.51–5.22).

Finally, parental cannabis use was the strongest predictor of adolescent cannabis use. Cannabis use by the mother increased the adolescent’s cannabis use by almost sevenfold (aOR = 6.78, 95% CI 1.42–32.38), and cannabis use by the father increased adolescent cannabis use by almost elevenfold (aOR = 10.93, 95% CI 3.50–34.11). Our full model explained 41.5% of variance (Nagelkerke) in adolescent lifetime prevalence of cannabis use.

## 4. Discussion

During adolescence, behaviors are formed that significantly impact an individual’s health and behavior in adulthood, including drug use [7,8,9,10,46]. A good understanding of the prevalence of the problem, the risk factors, and the protective factors associated with drug use is one of the keys to preventing the development of these behaviors [14,35,47].

In our study, we analyzed the effects of the mother’s and father’s parenting styles, cannabis-specific rules concerning the adolescent’s cannabis use, and parents’ cannabis use on the adolescent’s cannabis use. Several studies in Slovenia [3,4,5] show that drug use among adolescents is a public health issue and a wider social problem. Cannabis is the most commonly used illicit drug among adolescents in Slovenia [3,4], which places Slovenia above the international average on many indicators of cannabis use [3]. Use typically begins in adolescence and becomes most common in late adolescence and young adulthood [1]. Cannabis use is associated with an increased risk of dropping out of secondary school, not enrolling in university, failing to attain a degree, and other adverse adolescent outcomes [2,8,10,11,13,76].

Although cannabis is illegal in Slovenia and adolescents mostly use cannabis secretly and without parental knowledge or approval, parents play a key role in the adolescent’s initiation of cannabis use and subsequent patterns of use [2]. The results of our study show that 25.7% of young people included in the sample with a mean age of 16.43 years have used cannabis at least once in their lifetime, which is consistent with previous studies on the prevalence of cannabis use among adolescents with a comparable mean age in Slovenia [3,4].

The key findings of our study are that the mother’s permissive parenting style is a statistically significant predictor of adolescent cannabis use. However, maternal attitudes towards adolescent cannabis use and parental cannabis use have an even stronger impact on adolescent cannabis use. The findings of our study support those of previous research on the protective effect of the authoritative style on adolescent cannabis use [6,21,25]. Our results show that a mother’s authoritative parenting decreases the likelihood of adolescent cannabis use compared with the permissive style. This is consistent with previous research showing that permissive parenting increases the risk of adolescent cannabis use [21,27,77]. For paternal parenting styles, our research shows that compared with the permissive style, only authoritarian parenting increases the likelihood of an adolescent’s cannabis use, but not significantly. The results from the current study do not fully support previous research about the negative consequences of authoritarian parenting [25,77]. Although its negative impact did not reach statistical significance in the present study—most likely due to the exhaustive list of confounders in our model—our results nonetheless point to its negative role. Furthermore, considering the important role of parenting styles in numerous youth outcomes detected in the literature, the lack of significance of the majority of parenting styles in the present study with regard to adolescent cannabis use should not discourage parents to employ productive parenting styles (especially authoritative parenting).

Consistent with previous research [27,33,60,64,78], we also found that cannabis use among adolescents is associated with more tolerant parental attitudes towards cannabis use. However, the effect of the mother’s attitude was the only statistically significant one; if the mother does not have a markedly negative attitude towards the adolescent’s cannabis use, the probability of the adolescent using cannabis almost triples. Consistent with previous studies [33,60,61,62], we similarly found parental cannabis use to be a significant contributor to whether an adolescent ever used cannabis. Cannabis use by the mother increased this possibility by over sixfold and by almost elevenfold when the father uses cannabis, indicating that both of parental behaviors are the strongest predictors of adolescent cannabis use among Slovenian secondary school students.

Overall, we found that (1) authoritative maternal and paternal parenting styles are the most protective against adolescent cannabis use, although the maternal permissive style turned out to be the only significant parenting style risk factor; (2) parental cannabis use is the strongest risk factor for adolescent cannabis use, followed by the mother’s tolerant cannabis-specific rules, and the mother’s permissive parenting; (3) both parents play an important role in adolescent cannabis use: maternal parenting matters the most through parenting styles and cannabis-specific rules, while paternal parenting matters mainly in the form of role modelling related to cannabis use (i.e., the father not using cannabis), which has the strongest parental impact on adolescent cannabis use.

Adolescent drug use is a sensitive and complex area, influenced by the interplay of many factors, which need to be explored and understood, including those related to public health. From the perspective of public health promotion, our findings suggest that the presence of maternal authoritative style and the absence of permissive parenting style, strict parental (mother’s) disapproval of adolescent cannabis use, and parental non-use of cannabis are significant protective factors against adolescent cannabis use. Our study is consistent with findings from previous research [79] that maternal parenting style (compared with the father’s parenting) is a stronger determinant of adolescent outcomes, most likely because mothers tend to spend more time with their children [80]. However, it is the father’s cannabis use that is a somewhat stronger predictor of adolescent cannabis use than the mother’s cannabis use.

Cannabis use among adolescents usually starts in secret, without the parents’ knowledge. Once they become aware that their adolescent is using cannabis, the parents’ disapproval may not necessarily be sufficient; it is necessary to carefully plan preventive interventions and systemic measures aimed at preventing adolescents’ initial cannabis use. Our study suggests that the planning of preventive interventions should focus on parenting and parental influence and their role-modelling. This means that interventions should target the population of parents to strengthen their parenting competences, inform them about risky parenting styles, permissive attitudes toward drug use, and the very detrimental consequences of their drug use as role models [60,64]. Our results suggest that the importance of professionals working with parents should be emphasized, including in general practices (school doctors and nurses, pediatricians, helpline specialists, school counsellors, psychologists, etc.).

### Strenghts and Limitations of the Study and Suggestions for Future Research

The strength of the present study lies in its comparative analysis of the effect of family factors (parenting styles, cannabis-specific rules concerning adolescent cannabis use, and parental cannabis use) on adolescent cannabis use, with a separate analysis of the influence of mothers and fathers. The study is based on a large representative sample, so that the results can be generalized to the general population of Slovenian secondary school students. The prevalence of cannabis use in our study is similar to that of cannabis use among adolescents reported in national surveys [3,4]. However, future studies are needed to assess whether the study results can be generalized to young people from other western countries and non-western cultural environments. Among the study’s shortcomings, the collected survey data are based only on the adolescents’ self-reporting. However, several studies suggest that the method of self-reported drug use and parenting perception provides valid data [18,19,40,54]. Nevertheless, other data collection methods, e.g., interviews with parents, can prove useful in future research. Another limitation of the study is that our survey did not include school dropouts, who are among the most vulnerable because they belong to a high-risk group of young people [1]. Hence, future analyses of this subgroup of young people are also necessary. In addition, to compare the impact of maternal and paternal parenting styles, we examined only two-parent families, so future studies should investigate single-parent families also. Finally, we did not examine other potentially relevant factors that might impact cannabis use, including genetic predispositions [81], personality characteristics and parental mental health [15], and the quality of the family relationships, e.g., level of relationship conflict. All these factors may play an important role in determining the use of cannabis in adolescents and need to be examined in the future.

## 5. Conclusions

Our study showed that the authoritative and non-permissive parenting style of the mother, maternal disapproval of adolescent cannabis use, and parental (especially the father’s) non-use of cannabis are significant protective factors against adolescent cannabis use. In the context of effective prevention of adolescent cannabis use, it is essential to address parents and particularly their cannabis use and rules regarding adolescent cannabis use. Public health efforts should reflect the key goal of preventing today’s young people from becoming tomorrow’s drug users. Therefore, effective preventive efforts should be in place concerning adolescent drug use, particularly through the empowerment of parents and adolescents themselves. The present study contributes to existing literature by being the first to simultaneously assess three different dimensions of parental influence separately for the mother and the father, on adolescent cannabis use in a non-Western European country. The findings of the study can contribute to the development of public policies to prevent cannabis use among adolescents in Slovenia and in other countries worldwide.

## Figures and Tables

**Table 1:** Descriptive statistics of demographic, parenting, and confounding variables.

		N (%)	Lifetime Prevalence of Cannabis Use (%)	95% CI	Chi-Square Value
Overall		839	25.7%	22.8–28.8	/
Parenting variables					
Mother’s parenting styles	Authoritative	220 (26.2%)	13.6%	9.4–18.9	24.588 ***
	Authoritarian	208 (24.8 %)	26.9%	21.0–33.5	
	Permissive	245 (29.2%)	31.0%	25.3–37.2	
	Neglectful	166 (19.8%)	32.5%	25.5–40.2	
Father’s parenting styles	Authoritative	230 (27.4%)	16.1%	11.6–21.5	16.992 ***
	Authoritarian	227 (27.1%)	32.2%	26.1–38.7	
	Permissive	201 (24.0%)	28.4%	22.2–35.1	
	Neglectful	181 (21.6%)	27.1%	20.7–34.2	
Mother’s cannabis-specific rules	Does/would not allow	665 (79.3%)	21.4%	18.3–24.7	32.348 ***
	Other	174 (20.7%)	42.5%	35.1–50.2	
Father’s cannabis-specific rules	Does/would not allow	668 (79.6%)	22.2%	19.1–25.5	22.087 ***
	Other	171 (20.4%)	39.8%	32.4–47.5	
Mother’s cannabis use	Does not use	818 (97.5%)	24.3%	21.4–27.4	34.340 ***
	Uses	21 (2.5%)	81.0%	58.1–94.6	
Father’s cannabis use	Does not use	802 (95.6%)	23.3%	20.4–26.4	56.091 ***
	Uses	37 (4.4%)	78.4%	61.8–90.2	
Confounders					
Sex	Male	385 (45.9%)	23.6%	19.5–28.2	1.655
	Female	454 (54.1%)	27.5%	23.5–31.9	
AgeM = 16.43 (SD = 1.28)	14–15	225 (26.8%)	7.6%	4.5–11.8	77.158 ***
	16	230 (27.4%)	21.7%	16.6–27.6	
	17	209 (24.9%)	35.9%	29.4–42.8	
	18 and over	175 (20.9%)	42.3%	34.9–50.0	
Size of residential settlement	Rural settlement	383 (45.6%)	20.9%	16.9–25.3	9.516 **
	1000–50,000 inhabitants	282 (33.6%)	28.4%	23.2–34.0	
	More than 50,000 inhabitants	174 (20.7%)	32.2%	25.3–39.7	
Educational track	Vocational education	151 (18.0%)	14.6%	9.4–21.2	24.189 ***
	Technical education	427 (50.9%)	23.7%	19.7–28.0	
	General education	261 (31.1%)	35.6%	29.8–41.8	
Mother’s education	Vocational secondary or lower	217 (25.9%)	16.6%	11.9–22.2	20.228 ***
	Technical secondary or grammar school	300 (35.8%)	24.0%	19.3–29.2	
	College or higher	322 (38.4%)	33.5%	28.4–39.0	
Father’s education	Vocational secondary or lower	253 (30.2%)	23.7%	18.6–29.4	5.073
	Technical secondary or grammar school	319 (38.0%)	23.2%	18.7–28.2	
	College or higher	267 (31.8%)	30.7%	25.2–36.6	
Family material status	Below average	101 (12.0%)	31.7%	22.8–41.7	14.187 ***
	Average	506 (60.3%)	21.1%	17.7–25.0	
	Above average	232 (27.7%)	33.2%	27.2–39.7	
Self-rated health	Poor/fair	76 (9.1%)	31.6%	21.4–43.3	1.488
	Good/very good/excellent	763 (90.9%)	25.2%	22.1–28.4	
Academic achievement	Fail or satisfactory	287 (34.2%)	33.4%	28.0–39.2	13.545 ***
	(Very) good or excellent	552 (65.8%)	21.7%	18.4–25.4	
Alcohol use	Never	157 (18.7%)	1.9%	0.4–5.5	57.393 ***
	User	682 (81.3%)	31.2%	27.8–34.9	

Note: ** *p* < 0.01; *** *p* < 0.001.

**Table 2:** Association between the adolescents’ lifetime cannabis use, parenting practices and confounding variables.

Variables	Sig.	Exp (B)	95% CI for EXP (B)	
			Lower	Upper
Mother’s parenting style (reference: authoritative style)	0.162			
Neglectful	0.152	1.603	0.841	3.057
Authoritarian	0.179	1.536	0.821	2.874
Permissive	0.024	2.001	1.098	3.646
Father’s parenting style (reference: authoritative style)	0.331			
Neglectful	0.851	0.942	0.509	1.746
Authoritarian	0.222	1.436	0.803	2.569
Permissive	0.724	0.896	0.487	1.647
Mother’s cannabis-specific rules (reference: yes)	0.001	2.809	1.512	5.220
Father’s cannabis-specific rules (reference: yes)	0.588	0.842	0.452	1.569
Mother’s cannabis use (reference: no)	0.016	6.780	1.419	32.381
Father’s cannabis use (reference: no)	0.001	10.929	3.502	34.113
Sex (reference: male)	0.049	1.494	1.002	2.228
Age	0.001	2.051	1.689	2.490
Size of residential settlement	0.477	1.099	0.847	1.425
Educational track	0.004	1.681	1.183	2.386
Mother’s education	0.001	1.774	1.316	2.390
Father’s education	0.086	0.777	0.583	1.036
Family material status	0.855	1.030	0.751	1.413
Academic achievement (reference: high)	0.004	1.857	1.219	2.828
Alcohol use (reference: no)	0.001	36.421	8.762	151.388

## Data Availability

The data presented in this study are available upon a reasonable request from the corresponding author.
